# High Serum Estradiol Reduces Acute Hepatotoxicity Risk Induced by Epirubicin Plus Cyclophosphamide Chemotherapy in Premenopausal Women with Breast Cancer

**DOI:** 10.3389/fphar.2020.572444

**Published:** 2021-01-27

**Authors:** Shunmin Huang, Maobai Liu, Fangmeng Fu, Hangmin Liu, Baochang He, Danni Xiao, Jing Yang

**Affiliations:** ^1^ Department of Pharmacy, Fujian Medical University Union Hospital, Fuzhou, China; ^2^ School of Pharmacy, Fujian Medical University, Fuzhou, China; ^3^ Department of Breast Surgery, Fujian Medical University Union Hospital, Fuzhou, China; ^4^ Department of Clinical Laboratory, Fujian Medical University Union Hospital, Fuzhou, China; ^5^ Department of Epidemiology and Health Statistics, School of Public Health, Fujian Medical University, Fuzhou, China; ^6^ Department of Ultrasound, Fujian Medical University Union Hospital, Fuzhou, China

**Keywords:** breast neoplasms, drug-induced liver injury, adjuvant chemotherapy, estradiol, menopause

## Abstract

**Aim:** We evaluated whether acute drug-induced liver injury (DILI) caused by adjuvant chemotherapy with epirubicin plus cyclophosphamide for early breast cancer was associated with estradiol (E2), luteinizing hormone (LH), and follicle-stimulating hormone (FSH).

**Methods:** Reproductive hormone test results of breast cancer patients were collected in the first chemotherapy cycle. E2, LH, and FSH levels were log_e_-transformed to normally distributed variables and were assessed using Student’s *t*-test to determine significant differences between the case and control groups. Hormone levels were classified according to the interquartile range and analyzed by logistic regression to determine their association with DILI caused by chemotherapy.

**Results:** Among the 915 enrolled patients (DILI group: 204; control group: 711), menopausal status, along with serum E2, LH, and FSH levels, did not substantially differ between case and control groups. However, in the premenopause subgroup (n = 483), we found a significant difference in the E2 level between the case and control groups (*p* = 0.001). After adjusting for age and body mass index, premenopausal patients with 152–2,813 pg/mL E2 showed a lower risk of chemotherapy-induced DILI than patients with ≤20 pg/mL E2 (odds ratio: 0.394; 95% confidence interval: 0.207–0.748). The linear trend χ2 test revealed that E2 levels in premenopausal patients with breast cancer were inversely associated with the development of DILI.

**Conclusion:** High serum E2 levels are associated with a reduced DILI risk in premenopausal patients with breast cancer undergoing epirubicin plus cyclophosphamide adjuvant chemotherapy.

## Introduction

The EC-T regimen (epirubicin plus cyclophosphamide followed by docetaxel, every 3 weeks) is the first-line postoperative adjuvant chemotherapy treatment in patients with early breast cancer (Gradishar et al., 2020). In addition to eliminating tumor cells, these drugs have adverse effects on normal cells, tissues, and organs. Acute drug-induced liver injury (DILI) caused by chemotherapy limits the clinical use of the EC-T regimen, as DILI can result in illness, hospitalization, and even life-threatening liver failure, death, or the need for liver transplantation (Andrade et al., 2019).

The occurrence and severity of DILI associated with chemotherapy differs substantially between patients (Saithanyamurthi and Faust, 2017). Large-scale epidemiological surveys worldwide have confirmed that clinical factors, such as age, sex, non-alcoholic fatty liver disease, infection with hepatitis B virus, and underlying diseases, affect the occurrence of DILI (Sharma et al., 2002; Pukenyte et al., 2007; Stine and Lewis, 2011; Chalasani et al., 2014; Martinez et al., 2015). The average onset time of acute DILI by chemotherapy in breast cancer patients ranges from 6.2 to 17.9 days (Wang et al., 2012). Previous studies showed that women are more prone to experiencing DILI compared with men, and premenopausal patients tend to have a higher risk of DILI than postmenopausal patients (Andrade et al., 2005; Sgro et al., 2002; Wang et al., 2012; Suzuki et al., 2017), but the underlying mechanism remains unclear.

Sex and hormones may influence drug metabolism and transport and modulate host responses to injury (Beagley and Gockel, 2003; Waxman and Holloway, 2009; Yang et al., 2012). A study using an immune-mediated DILI model demonstrated sex differences in the immune response and inflammation; female mice elicited more vigorous cellular and humoral immune reactions (Cho et al., 2013). Importantly, the immune system is regulated by circulating levels of sex hormones (Grossman, 1985). For example, estradiol (E2) inhibited the production of proinflammatory cytokines in mouse peritoneal macrophages and monocytes *in vitro* (Yuan et al., 2008). Furthermore, in a mouse model, estrogen reduced the risk of halothane-induced liver injury (Toyoda et al., 2011). Thus, we hypothesized that the level of estrogen may affect the development of DILI caused by chemotherapy in patients with breast cancer. However, there are no validated estrogen biomarkers to identify the predisposition of female patients with breast cancer receiving chemotherapy to develop DILI.

In this study, we investigated the relationship between the estrogen level and acute DILI in patients receiving the first cycle of EC-T adjuvant chemotherapy. We performed an in-depth analysis of important clinical and pathophysiological questions relevant to DILI in pre- and postmenopausal patients with breast cancer. Identifying the effect of estrogen on DILI may contribute to the development of improved individual treatment options for patients with breast cancer.

## Material and Methods

### Patients

**FIGURE 1:**
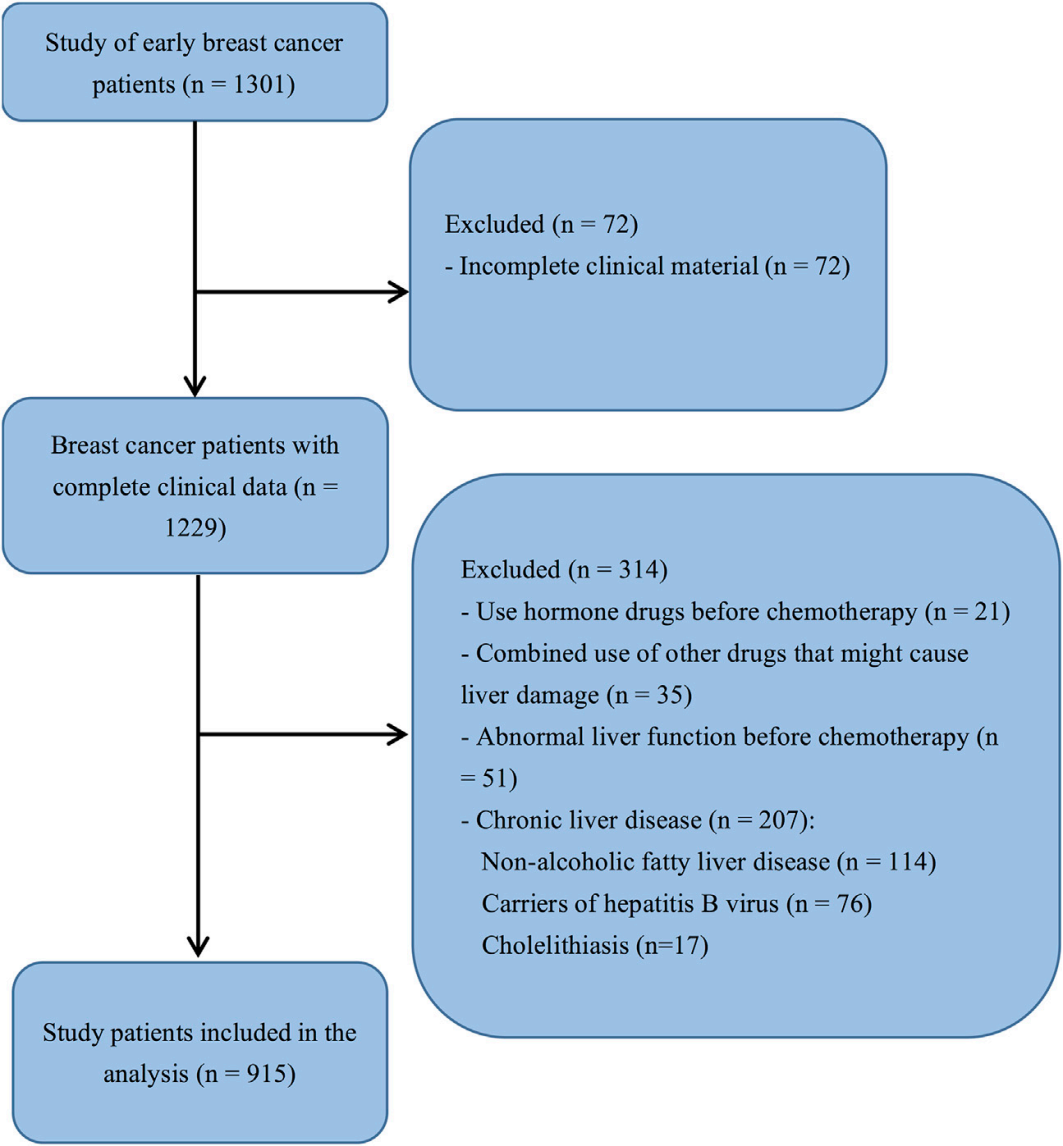
Flowchart of study participant selection. The analysis included all patients with complete clinical data (n = 1,229). After exclusions, the final sample size was 915 patients.

This retrospective case-control study included female patients with breast cancer who were administered adjuvant chemotherapy between September 2014 and January 2018 at the Fujian Medical University Union Hospital (Fuzhou, China). Patients who received EC-T chemotherapy in the first cycle and developed liver injury were included in the case group, whereas patients who did not develop liver toxicity during the first cycle of EC-T were included in the control group. Menopause was defined as the absence menstrual cycle for 12 consecutive months. The inclusion criteria were as follows: (a) patients with breast cancer at pathological stage Ib-II, (b) patients with an estrogen test during the first cycle of chemotherapy, and (c) a postoperative adjuvant chemotherapy regimen with epirubicin plus cyclophosphamide followed by docetaxel. The exclusion criteria were as follows: (a) incomplete clinical material, (b) administration of hormone drugs before chemotherapy, (c) combined administration of other drugs that may cause liver damage, (d) abnormal liver function before the first chemotherapy cycle, and (e) prior diagnosis of chronic liver diseases such as non-alcoholic fatty liver disease, cholelithiasis, and carriers of hepatitis B virus. The final sample size was 915 patients with early breast cancer (Figure 1).

We collected details related to the demographic characteristics, accompanying diseases, and combined medication information of the study participants. We also collected information on the biochemical indices of liver function before initial chemotherapy and after the first cycle of chemotherapy, including alanine aminotransferase (ALT), aspartate aminotransferase (AST), alkaline phosphatase (ALP), and total bilirubin. The patients’ in-hospital information was derived from the hospital medical records information management system, and outpatient information was obtained through face-to-face, telephone, or WeChat (the most popular messaging app in China) follow-up interviews. The ethics committee of Fujian Medical University Union Hospital approved the study, and all patients provided informed consent for their participation. The study was performed in accordance with The Code of Ethics of the 1964 Declaration of Helsinki and its later amendments.

### Measurements of Reproductive Hormones

Estrogen levels were tested one day before administration of the first cycle of adjuvant chemotherapy. Overnight-fasting venous blood samples were collected between 08:00 and 09:00 h after the subjects had rested for 20–30 min. The blood samples were placed in tubes without anticoagulant and then centrifuged at 100 × *g* for 15 min to separate the serum from the blood. The serum levels of E2, LH, and FSH were measured with an ACCESS Automated Chemiluminescent Immunoassay System (Beckman Coulter, Brea, CA, USA).

### Definition of DILI Associated With Chemotherapy

This study focused on the first cycle of chemotherapy. The case definition for DILI included one of the following thresholds, according to the Guide to Drug-induced Liver Injury (Aithal et al., 2011): (i) ≥5 upper limit of normal (ULN) elevation in ALT, (ii) ≥2 ULN elevation in ALP (specifically with an accompanying elevated level of gamma-glutamyltransferase in the absence of bone pathologies, which is known to promote an increase in the ALP level), or (iii) ≥3 ULN elevation in ALT and simultaneous ≥2 ULN elevation in total bilirubin.

### Statistical Analyses

Data analyses were performed using SPSS 20.0 software (IBM, Chicago, IL, USA). Normally distributed continuous variables were expressed as means ± SD and compared by DILI status using Student’s *t*-test. Variables with skewed distributions were expressed as medians (interquartile range) and compared using the nonparametric Wilcoxon rank-sum test. E2, LH, and FSH levels were log_e_-transformed to normally distributed variables. Categorical variables were expressed as percentages and compared using the chi-square test. The continuous variable, estrogen, was reported as quartile intervals and transformed into a categorical variable. Postmenopausal women typically have lower E2 levels; in the current study, approximately 70% had <20 pg/ml. The stratification criteria of E2 for postmenopausal patients were ≤P75 (interquartile range 75) and >P75. The linear trend χ2 test was used to analyze the trend of DILI risk with the estrogen level. The association between different estrogen levels and DILI was determined by a logistic regression analysis, adjusted for age and body mass index (BMI). Owing to the many stratified levels of estrogen assessed, corrections for multiple hypothesis testing were performed to preserve the overall type I error rate by calculating the false discovery rate for each of the stratified levels of E2, LH, and FSH assessed. All statistical tests were two-sided probability tests with a significance level of *α* = 0.05.

## Results

The clinical characteristics of the study population at enrollment are summarized in Table 1. A total of 915 patients (premenopausal group: 483; postmenopausal group: 432) with breast cancer were included in the study. Overall, 204 (22.3%) patients were diagnosed with DILI. These patients had a mean age of 49.4 ± 9.4 years (range: 22–66) and mean BMI of 22.7 ± 2.9 (range: 16.7–30.5). The mean age of patients not diagnosed with DILI was 48.3 ± 8.5 years (range: 25–82), and their mean BMI was 23.0 ± 2.9 (range: 16.7–33.2). The case and control groups did not substantially differ in age and BMI or regarding the serum levels of E2, LH, and FSH.

**TABLE 1:** Characteristics of patients with and without drug-induced liver injury caused by chemotherapy.

Incident DILI status	Patients without DILI (n = 711)	Patients with DILI (n = 204)	*p*
Age	48.3 ± 8.5	49.4 ± 9.4	0.123
BMI	23 ± 2.9	22.7 ± 2.9	0.266
Postmenopausal, n (%)	344 (48.4)	88 (43.1)	0.186
E2 (pg/ml)	3.42 ± 1.32	3.33 ± 1.08	0.379
LH (mIU/ml)	2.58 ± 1.22	2.53 ± 1.27	0.609
FSH (mIU/ml)	3.18 ± 1.35	3.15 ± 1.31	0.759
Laboratory parameters			
ALT (IU/L), median (25–75%)	30 (21–64)	107 (78–156)	<0.001
AST (IU/L), median (25–75%)	29 (23–57)	112 (76–144)	<0.001
ALP (IU/L), median (25–75%)	84 (57–90)	141 (90–189)	<0.001
TBIL (μmol/L), median (25–75%)	5.5 (2.1–9.0)	14.2 (5.6–23.3)	<0.001

All sex hormone levels are log_e_-transformed and standardized. DILI, drug-induced liver injury; BMI, body mass index; E2, estradiol; LH, luteinizing hormone; FSH, follicle-stimulating hormone; TBIL, total bilirubin. Data are expressed as means ± SD.

The results of a stratified analysis of associations between DILI and the serum values of E2, LH, and FSH are shown in Tables 2–4. In the premenopausal subgroup, there was a significant difference in the E2 level between the case and control groups (*p* = 0.001) (Table 2). A higher E2 level was independently associated with a decreased risk of incident DILI in premenopausal women (Table 3). The fully adjusted odds ratio for DILI associated with a one standard deviation higher log-transformed E2 level was 0.782 (95% confidence interval: 0.656–0.933). In the postmenopausal subgroup, age differed substantially between the case and control groups, with mean ages ±standard deviation of 57.2 ± 5.0 and 55.6 ± 3.8 years (*p* = 0.001), respectively.

**TABLE 2:** Distribution of estrogen in premenopausal and postmenopausal subgroups.

	Premenopause		*p*	Postmenopause		*p*
	Patients without DILI (n = 367)	Patients with DILI (n = 116)		Patients without DILI (n = 344)	Patients with DILI (n = 88)	
Age	42.1 ± 6.0	42.8 ± 6.8	0.318	55.6 ± 3.8	57.2 ± 5.0	**0.001**
BMI	23.1 ± 2.9	22.8 ± 2.9	0.361	22.9 ± 2.8	22.6 ± 2.8	0.469
E2 (pg/ml)	4.13 ± 1.27	3.69 ± 1.15	**0.001**	2.67 ± 0.87	2.87 ± 0.77	0.057
LH (mIU/ml)	2.00 ± 1.30	1.96 ± 1.29	0.75	3.19 ± 0.74	3.28 ± 0.75	0.33
FSH (mIU/ml)	2.36 ± 1.30	2.48 ± 1.25	0.382	4.06 ± 0.69	4.04 ± 0.73	0.839

All sex hormone levels are log_e_-transformed and standardized. *p* values in **bold font** indicate statistically significant results (*p* < 0.05). DILI, drug-induced liver injury; E2, estradiol; LH, luteinizing hormone; FSH, follicle-stimulating hormone. Data are expressed as means ± SD.

**TABLE 3:** Association of DILI with estrogen levels (per one standard deviation) in premenopausal and postmenopausal patients with breast cancer.

Study group	OR (95% CI)		
	Model 1	Model 2	Model 3
Premenopause			
E2 (pg/ml)	0.782 (0.656–0.932)	**0.777 (0.654–0.922)**	**0.782 (0.656–0.933)**
LH (mIU/ml)	0.940 (0.791–1.115)	0.973 (0.828–1.143)	0.940 (0.792–1.116)
FSH (mIU/ml)	1.045 (0.873–1.251)	1.071 (0.910–1.260)	1.043 (0.871–1.249)
Postmenopause			
E2 (pg/ml)	1.118 (0.809–1.544)	1.123 (0.904–1.578)	1.125 (0.813–1.555)
LH (mIU/ml)	1.212 (0.860–1.708)	1.191 (0.836–1.697)	1.213 (0.862–1.706)
FSH (mIU/ml)	1.051 (0.750–1.474)	0.965 (0.694–1.342)	1.054 (0.752–1.478)

All sex hormone levels are log_e_-transformed and standardized. Results in **bold font** are statistically significant (*p* < 0.05). BMI, body mass index; DILI, drug-induced liver injury; E2, estradiol; LH, luteinizing hormone; FSH, follicle-stimulating hormone; OR, odds ratio; CI, confidence interval. Model one adjusts for age. Model two adjusts for BMI. Model three adjusts for age and BMI.

**TABLE 4:** Analysis of estrogen levels associated with DILI in premenopausal and postmenopausal patients.

Study group	Patients without DILI (n)	Patients with DILI (n)	Linear trend χ^2^	*P*_*1*_	OR^a^ (95% CI)	*P*_*2*_ ^a^	FDR
Premenopause							
E2 (pg/ml)			0.838	**0.003**			
≤20	82	38			Ref		
20–61	93	32			0.780 (0.437–1.391)	0.294	0.672
61–152	90	28			0.695 (0.385–1.254)	0.173	0.723
152–2,813	102	18			**0.394 (0.207–0.748)**	**0.003**	**0.048**
LH (mIU/ml)			0.001	0.973			
≤3.41	91	30			Ref		
3.41–7.14	90	31			1.028 (0.573–1.844)	0.882	0.882
7.14–19.2	99	22			0.643 (0.342–1.209)	0.212	0.678
19.2–83.7	87	33			1.042 (0.564–1.925)	0.633	0.779
FSH (mIU/ml)			1.619	0.203			
≤4.27	97	24			Ref		
4.27–8.01	92	29			1.240 (0.671–2.292)	0.438	0.779
8.01–34.5	90	31			1.319 (0.709–2.455)	0.284	0.757
34.5–141	88	32			1.355 (0.703–2.610)	0.211	0.844
Postmenopause							
E2 (pg/ml)			0.076	0.783			
≤22	259	65			Ref		
22–537	85	23			1.037 (0.603–1.785)	0.783	0.835
LH (mIU/ml)			0.923	0.337			
≤19.1	86	22			Ref		
19.1–28.2	89	19			0.907 (0.453–1.813)	0.603	0.965
28.2–37.8	89	19			0.830 (0.417–1.652)	0.603	0.877
37.8–114	80	28			1.298 (0.682–2.470)	0.334	0.668
FSH (mIU/ml)			0.011	0.915			
≤49.2	83	25			Ref		
49.2–67.0	86	22			1.011 (0.519–1.971)	0.621	0.828
67.0–84.6	94	14			0.546 (0.264–1.131)	0.054	0.432
84.6–203	81	27			1.177 (0.625–2.217)	0.75	0.857

*p* values in **bold font** indicate statistically significant results. DILI, drug-induced liver injury; E2, estradiol; LH, luteinizing hormone; FSH, follicle-stimulating hormone; Ref, reference; OR, odds ratio; CI, confidence interval; *P*_*1*_, *p* values from linear trend χ^2^ analyses; *P*_*2*_, *p* values from unconditional logistic regression analyses;

^a^ Logistic regression with adjustment for age and body mass index; FDR, false discovery rate.

The linear trend χ^2^ test showed that the E2 levels of patients with and without DILI were correlated in the premenopausal subgroup; the linear trend χ^2^ was 8.838 (*p* = 0.003) (Table 4). After adjusting for age and BMI, premenopausal patients with an E2 level of 152–2,813 pg/ml showed a lower risk of DILI than those with the E2 level ≤20 pg/ml (odds ratio: 0.394; 95% confidence interval: 0.207–0.748, *p*: 0.003). After correction for multiple hypothesis testing, the association with an E2 level of 152–2,813 pg/ml remained significant (false discovery rate = 0.048). However, among other patients with breast cancer, DILI was not associated with the serum levels of E2, LH, or FSH.

## Discussion

The EC-T regimen for patients with early breast cancer may lead to acute liver injury by increasing the reactive oxygen species level, which leads to the generation of oxidative stress and disrupts cellular homeostasis (Henninger et al., 2012; Li et al., 2014; Li et al., 2020). The hepatotoxicity of adjuvant chemotherapy limits its use in patients with cancer. Although some studies have been performed on the effects of menopausal status on DILI (Wang et al., 2012; Suzuki et al., 2017), whether sex hormones affect DILI in patients with early breast cancer remains unknown, as it is difficult to obtain a large number of patients with hormone tests. In this study, we collected estrogen data from patients with breast cancer before the first cycle of adjuvant chemotherapy and explored the correlation between estrogen levels and DILI caused by chemotherapy.

To accurately assess the effect of estrogen levels on DILI, we excluded patients with risk factors for DILI such as hepatitis B virus carriers, cholelithiasis, and non-alcoholic fatty liver disease. We finally assigned 204 cases to the case group and 711 cases to the control group. The distribution of menopausal status and estrogen levels did not substantially differ between the case and control groups. This is inconsistent with the results of a previous study showing that the menopausal status influenced DILI in patients with metastatic breast cancer (Wang et al., 2012). This may be related to the exclusion criteria of the current study. Hepatitis B virus carriers, cholelithiasis, and fatty liver identified as underlying liver diseases may increase the susceptibility to DILI (Lewis et al., 2007; Massart et al., 2017; Sawada et al., 2019).

Interestingly, after subgrouping the 915 patients with breast cancer according to their menopausal status, the E2 levels differed significantly between the case (116 patients) and control (367 patients) groups. The linear trend χ2 test revealed that E2 levels in premenopausal patients with breast cancer were inversely associated with the development of DILI. Indeed, the risk of DILI was lower in patients with breast cancer who received adjuvant chemotherapy and had higher levels of E2. This is the first study to show that high E2 levels decreased the risk of acute DILI caused by EC-T regimen adjuvant chemotherapy in premenopausal patients with breast cancer. However, the protective mechanism of E2 on DILI has not been studied in humans. In addition, we found that age may be a factor for DILI induced by EC-T regimen adjuvant chemotherapy in postmenopausal patients with breast cancer.

Although the liver is not a classic sex hormone target, it contains estrogen receptors and responds to estrogen (Eagon et al., 1991; Villa et al., 1995). After binding to the estrogen receptor in the liver, estrogen may regulate the synthesis of biomolecules related to liver injury that can affect the occurrence of DILI. The protective mechanism of E2 on DILI has been demonstrated in animal experiments. Toyoda et al. (2011) found that pretreatment of mice with E2 attenuated halothane-induced liver injury. This group observed that lower numbers of neutrophils infiltrated the liver, and there were decreased hepatic mRNA levels of proinflammatory cytokines (including tumor necrosis factor and interleukin-1 and -6) and chemokines (CXCL1 and CXCL2). Zhang and colleagues (Zhang et al., 2012) discovered that E2 inhibited carbon tetrachloride-induced hepatic injury in mice by inducing the expression of miRNA-29 (including the key collagen regulators miR-29a and miR-29b), which is implicated in tissue fibrosis (Maurer et al., 2010; Roderburg et al., 2011). Hence, the underlying mechanisms of E2-induced biomolecular changes affecting DILI in animal experiments may also play a critical role in patients with breast cancer experiencing DILI caused by chemotherapy.

Our study had some limitations. Serum estrogen levels in premenopausal women fluctuate during the physiological cycle. Therefore, we only conducted a correlation study between liver injury after the first cycle of chemotherapy and the level of estrogen one day before chemotherapy. In the future, we intend to do a more comprehensive and detailed analysis of the relationship between estrogen and DILI in later cycles of EC-T chemotherapy.

In summary, we demonstrated that E2 was significantly associated with DILI in premenopausal patients with breast cancer. The results of this study can provide guidance for effective prevention and treatment of DILI caused by adjuvant chemotherapy for breast cancer in patient populations that differ in their menopausal status. However, the molecular mechanism of serum estrogen protection against DILI in patients with breast cancer remains unclear. Further clinical analyses and confirmation by experimental studies are needed to understand the role of estrogen in hepatotoxicity and DILI manifestations.

## Data Availability Statement

The raw data supporting the conclusions of this article will be made available by the authors, without undue reservation.

## Ethics Statement

The studies involving human participants were reviewed and approved by The ethics committee of Fujian Medical University Union Hospital approved the study, and all patients provided informed consent for their participation. The patients/participants provided their written informed consent to participate in this study.

## Author Contributions

Data analysis: SH, BH, HL, and DX. Manuscript preparation: SH, JY, ML, and FF. Critical revision of the manuscript: JY and ML. Study supervision: JY and FF.

## Funding

SH received a research grant from the Startup Fund for Scientific Research, Fujian Medical University (Grant No. 2017XQ1025). JY received research grants from Joint Funds for the Innovation of Science and Technology, Fujian Province (Grant No. 2018Y9045) and the Key Project for Youth Academic Talents from the Health and Family Planning Commission of Fujian Province (Grant No. 2019-ZQN-39).

## Conflict of Interest

The authors declare that the research was conducted in the absence of any commercial or financial relationships that could be construed as a potential conflict of interest.

## References

[B1] Aithal G. P. Watkins P. B. Andrade R. J. Larrey D. Molokhia M. Takikawa H. (2011). Case definition and phenotype standardization in drug-induced liver injury. Clin. Pharmacol. Ther. 89 (6), 806–815. 10.1038/clpt.2011.58 21544079

[B2] Andrade R. J. Lucena M. I. Fernández M. C. Pelaez G. Pachkoria K. García-Ruiz E. (2005). Drug-induced liver injury: an analysis of 461 incidences submitted to the Spanish registry over a 10-year period. Gastroenterology 129 (2), 512–521. 10.1016/j.gastro.2005.05.006 16083708

[B3] Andrade R. J. Aithal G. P. Björnsson E. S. Kaplowitz N. Kullak-Ublick G. A. Larrey D. (2019). EASL clinical practice guidelines: drug-induced liver injury. J. Hepatol. 70 (6), 1222–1261. 10.1016/j.jhep.2019.02.014 30926241

[B4] Beagley K. W. Gockel C. M. (2003). Regulation of innate and adaptive immunity by the female sex hormones oestradiol and progesterone. FEMS Immunol. Med. Microbiol. 38 (1), 13–22. 10.1016/S0928-8244(03)00202-5 12900050

[B5] Chalasani N. P. Hayashi P. H. Bonkovsky H. L. Navarro V. J. Lee W. M. Fontana R. J. (2014). ACG Clinical Guideline: the diagnosis and management of idiosyncratic drug-induced liver injury. Am. J. Gastroenterol. 109 (7), 950–967. 10.1038/ajg.2014.131 24935270

[B6] Cho J. Kim L. Li Z. Rose N. R. Talor M. V. Njoku D. B. (2013). Sex bias in experimental immune-mediated, drug-induced liver injury in BALB/c mice: suggested roles for tregs, estrogen, and IL-6. PloS One 8 (4), e61186 10.1371/journal.pone.0061186 23577207 PMC3618451

[B8] Eagon P. K. Francavilla A. DiLeo A. Elm M. S. Gennari L. Mazzaferro V. (1991). Quantitation of estrogen and androgen receptors in hepatocellular carcinoma and adjacent normal human liver. Dig. Dis. Sci. 36 (9), 1303–1308. 10.1007/BF01307527 1654243

[B9] Gradishar W. J. Anderson B. O. Abraham J. Aft R. Agnese D. Allison K. H. (2020). Breast cancer, version 3.2020, NCCN clinical practice guidelines in oncology. J. Natl. Compr. Canc. Netw. 18, 452 10.6004/jnccn.2020.0016 32259783

[B10] Grossman C. J. (1985). Interactions between the gonadal steroids and the immune system. Science 227 (4684), 257–261. 10.1126/science.3871252 3871252

[B11] Henninger C. Huelsenbeck J. Huelsenbeck S. Grösch S. Schad A. Lackner K. J. (2012). The lipid lowering drug lovastatin protects against doxorubicin-induced hepatotoxicity. Toxicol. Appl. Pharmacol. 261 (1), 66–73. 10.1016/j.taap.2012.03.012 22712078

[B12] Lewis J. H. Mortensen M. E. Zweig S. Fusco M. J. Medoff J. R. Belder R. (2007). Efficacy and safety of high-dose pravastatin in hypercholesterolemic patients with well-compensated chronic liver disease: results of a prospective, randomized, double-blind, placebo-controlled, multicenter trial. Hepatology 46 (5), 1453–1463. 10.1002/hep.21848 17668878

[B13] Li A. Li S. Zhang Y. Xu X. Chen Y. Li H. (2014). Resources and biological activities of natural polyphenols. Nutrients 6 (12), 6020–6047. 10.3390/nu6126020 25533011 PMC4277013

[B14] Li B. Li W. Tian Y. Guo S. Qian L. Xu D. (2020). Selenium-alleviated hepatocyte necrosis and DNA damage in cyclophosphamide-treated geese by mitigating oxidative stress. Biol. Trace Elem. Res. 193 (2), 508–516. 10.1007/s12011-019-01717-3 31025241

[B16] Martinez M. A. Vuppalanchi R. Fontana R. J. Stolz A. Kleiner D. E. Hayashi P. H. (2015). Clinical and histologic features of azithromycin-induced liver injury. Clin. Gastroenterol. Hepatol. 13 (2), 369–e3. 10.1016/j.cgh.2014.07.054 25111234 PMC4321982

[B17] Massart J. Begriche K. Moreau C. Fromenty B. (2017). Role of nonalcoholic fatty liver disease as risk factor for drug-induced hepatotoxicity. J. Clin. Transl. Res. 3 (1), 212 10.18053/jctres.03.2017S1.006 28691103 PMC5500243

[B18] Maurer B. Stanczyk J. Jüngel A. Akhmetshina A. Trenkmann M. Brock M. (2010). MicroRNA-29, a key regulator of collagen expression in systemic sclerosis. Arthritis Rheum. 62 (6), 1733–1743. 10.1002/art.27443 20201077

[B20] Pukenyte E. Lescure F. X. Rey D. Rabaud C. Hoen B. Chavanet P. (2007). Incidence of and risk factors for severe liver toxicity in HIV-infected patients on anti-tuberculosis treatment. Int. J. Tubercul. Lung Dis. 11 (1), 78–84. 10.1016/j.hrtlng.2006.09.003 17217134

[B21] Roderburg C. Urban G. Bettermann K. Vucur M. Zimmermann H. Schmidt S. (2011). Micro-RNA profiling reveals a role for miR-29 in human and murine liver fibrosis. Hepatology 53 (1), 209–218. 10.1002/hep.23922 20890893

[B22] Saithanyamurthi H. Faust A. J. (2017). Drug-induced liver disease: clinical course. Clin. Liver Dis. 21 (1), 21–34. 10.1016/j.cld.2016.08.007 27842773

[B23] Sawada K. Hayashi H. Nakajima S. Hasebe T. Fujiya M. Okumura T. (2019). Non‐alcoholic fatty liver disease is a potential risk factor for liver injury caused by immune checkpoint inhibitor. J. Gastroenterol. Hepatol. 10.1111/jgh.14889 31752049

[B24] Sgro C. Clinard F. Ouazir K. Chanay H. Allard C. Guilleminet C. (2002). Incidence of drug-induced hepatic injuries: a French population-based study. Hepatology 36 (2), 451–455. 10.1053/jhep.2002.34857 12143055

[B25] Sharma S. K. Balamurugan A. Saha P. K. Pandey R. M. Mehra N. K. (2002). Evaluation of clinical and immunogenetic risk factors for the development of hepatotoxicity during antituberculosis treatment. Am. J. Respir. Crit. Care Med. 166 (7), 916–919. 10.1164/rccm.2108091 12359646

[B26] Stine J. G. Lewis J. H. (2011). Drug-induced liver injury: a summary of recent advances. Expet Opin. Drug Metabol. Toxicol. 7 (7), 875–890. 10.1517/17425255.2011.577415 21510822

[B27] Suzuki A. Barnhart H. Gu J. Bonkovsky H. L. Tillmann H. L. Fontana R. J. (2017). Associations of gender and a proxy of female menopausal status with histological features of drug-induced liver injury. Liver Int. 37 (11), 1723–1730. 10.1111/liv.13380 28161910 PMC5545077

[B28] Toyoda Y. Miyashita T. Endo S. Tsuneyama K. Fukami T. Nakajima M. (2011). Estradiol and progesterone modulate halothane-induced liver injury in mice. Toxicol. Lett. 204 (1), 17–24. 10.1016/j.toxlet.2011.03.031 21501669

[B29] Villa E. Camellini L. Dugani A. Zucchi F. Grottola A. Merighi A. (1995). Variant estrogen receptor messenger RNA species detected in human primary hepatocellular carcinoma. Canc. Res. 55 (3), 498–500. 10.1007/BF01520296 7834616

[B30] Wang Z. Liang X. Yu J. Zheng X. Zhu Y. Yan Y. (2012). Non-genetic risk factors and predicting efficacy for docetaxel--drug-induced liver injury among metastatic breast cancer patients. J. Gastroenterol. Hepatol. 27 (8), 1348–1352. 10.1111/j.1440-1746.2012.07131.x 22432938

[B31] Waxman D. J. Holloway M. G. (2009). Sex differences in the expression of hepatic drug metabolizing enzymes. Mol. Pharmacol. 76 (2), 215–228. 10.1124/mol.109.056705 19483103 PMC2713118

[B32] Yang L. Li Y. Hong H. Chang C. W. Guo L. W. Lyn-Cook B. (2012). Sex differences in the expression of drug-metabolizing and transporter genes in human liver. J. Drug Metabol. Toxicol. 3 (3). 10.4172/2157-7609.1000119 PMC5699760 29177108

[B33] Yuan Y. Shimizu I. Shen M. Aoyagi E. Takenaka H. Itagaki T. (2008). Effects of estradiol and progesterone on the proinflammatory cytokine production by mononuclear cells from patients with chronic hepatitis C. World J. Gastroenterol. 14 (14), 2200–2207. 10.3748/wjg.14.2200 18407594 PMC2703845

[B34] Zhang Y. Wu L. Wang Y. Zhang M. Li L. Zhu D. (2012). Protective role of estrogen-induced miRNA-29 expression in carbon tetrachloride-induced mouse liver injury. J. Biol. Chem. 287 (18), 14851–14862. 10.1074/jbc.M111.314922 22393047 PMC3340269

